# Limited association between liver stiffness and clinical outcomes in Fontan-associated liver disease: a retrospective analysis

**DOI:** 10.1016/j.ijcchd.2026.100685

**Published:** 2026-05-26

**Authors:** Louise Cai, Preeti Choudhary, David Tanous, Jacob George

**Affiliations:** a University of Sydney, Sydney, NSW, 2006, Australia; b Department of Cardiology, Westmead Hospital, Westmead, NSW, 2145, Australia; c Department of Gastroenterology and Hepatology, Westmead Hospital, Westmead, NSW, 2145, Australia; d Storr Liver Centre, Department of Medicine, Westmead Hospital, Westmead, NSW, 2145, Australia

## Abstract

The Fontan procedure is a palliative operation for patients with univentricular physiology. Liver complications post-surgery are inevitable and include liver cirrhosis and hepatocellular carcinoma (HCC). Routine surveillance includes transient elastography (TE) and clinical assessment, however longitudinal data on Fontan-associated liver disease (FALD) is scarce. This study aimed to describe the evolution of FALD and assess the correlation between liver stiffness measurement (LSM) and hepatic decompensation.

This retrospective case series comprised all adult post-Fontan patients presenting to a tertiary hospital between January 2015 and December 2024. Demographic and clinical information, serum tests, TE and echocardiograms were extracted from medical records. Endpoints assessed were decompensated cirrhosis and findings that warrant HCC multi-disciplinary team review. A derived surrogate outcome, clinically significant portal hypertension (CSPH) was assessed using the Baveno VII criteria.

There were a total of 65 patients with 111 Fibroscans™. There was no significant change in LSM by TE when comparing two consecutive decade years post Fontan surgery. On echocardiography, only inferior vena-cava diameter and degree of ventricular function correlated with LSM scores (p = 0.032, p < 0.001). Twelve patients had CSPH, correlating with increased gamma-glutamyltransferase, bilirubin, international normalised ratio and aspartate aminotransferase levels (p = 0.047, 0.034, 0.012, 0.049 respectively). Four patients exhibited features of decompensated cirrhosis, eight warranted multi-disciplinary team discussion however these did not correlate with LSM or steatosis results.

LSM and echocardiogram monitoring alone is insufficient for identifying FALD patients at risk of hepatic decompensation. Regular testing and multicenter research is required to develop predictive models that accurately identifies these at-risk patients.

## 1 Introduction

The Fontan procedure is a palliative operation for patients born with hypoplasia of one of the cardiac ventricles [1]. By directing the systemic venous return into the pulmonary circulation and minimising intra-cardiac mixing, the procedure elevates arterial oxygen saturation and decreases the chronic volume overload experienced by the single ventricle [2]. Advancements in surgical techniques, coupled with improving paediatric management of these patients, has increased the 30-year survival rate post-Fontan surgery to over 80% [3], allowing many patients to reach adulthood [2]. As a result, the adult post-Fontan population is growing by approximately 5% annually. This longer survival has heralded the emergence of both long-term cardiac and extra-cardiac complications [2].

Fontan-associated liver disease (FALD) is considered inevitable post-surgery [4] as nearly all patients develop hepatic congestion, potentially resulting in fibrosis and cirrhosis [5]. FALD is a distinct type of congestive hepatopathy with a multifactorial pathogenesis. From birth, the liver is exposed to chronic cyanosis and hypoxia, and staged cardiac interventions may further compound these haemodynamic insults [2,6]. Post-Fontan surgery, there is increased central venous pressure, low cardiac output and obstructed lymphatic outflow [5]. Combined, these mechanisms have deleterious effects on the liver, making FALD a growing concern. Ultimately, there is a risk of hepatic decompensation and transformation to cancer, most notably hepatocellular carcinoma, with an estimated prevalence rate of 0.18-1.3% [5].

Early stage FALD is clinically indolent [2,6]. Patients with compensated cirrhosis may be asymptomatic or present with non-specific symptoms such as weight loss or lethargy and symptoms such as encephalopathy or ascites may only intervene in advanced FALD [5]. Currently, early and accurate diagnosis of FALD remains challenging [7]. Liver biopsy, the gold standard in identifying FALD, may be inaccurate due to the heterogenous nature of the liver pathology and biopsy is infrequently performed due to the increased risk of bleeding from a high central venous pressure or anticoagulation [5].

Non-invasive techniques that have been validated for other liver diseases are being used to stage FALD, although these also have limitations [5]. Liver congestion in FALD can present with confounding features on transient elastography (TE), leading to false positive results [1,8]. Furthermore, blood markers of liver function often do not correlate with the severity of fibrosis or relate to clinical outcomes [6,7,9]. Nevertheless, the routine surveillance of patients following Fontan surgery often includes a combination of regular clinical assessment, laboratory tests, transient elastography, and radiological imaging to screen for fibrosis and hepatocellular carcinoma [5,10].

There is suboptimal longitudinal data on the manifestations and outcomes of FALD. This study aimed to describe the natural history of FALD in an adult cohort and to assess the utility of elastography in identifying clinically significant hepatic outcomes. Haemodynamic and functional performance of the Fontan circulation was assessed.

## 2 Methods

### 2.1 Design

This study is a retrospective analysis of all adult post-Fontan patients presenting to the liver service at a tertiary Australian hospital between January 2015 and December 2024. All patients over 14 years old (the criterion at the adult hospital for patients) in the catchment area were routinely referred by the adult congenital cardiac clinic for surveillance. Ethics approval was obtained from the Western Sydney Local Health District Human Research Ethics Committee (2019/ETH10751).

### 2.2 Data collection

Basic demographic details such as sex, date of birth, co-morbidities and medications were collected. The cardiac history collected include primary morphological diagnosis, Fontan procedure variant and age at Fontan procedure. Transient elastography data collected include time from Fontan to Fibroscan™, median liver stiffness measurement (LSM) and median hepatic controlled attenuation parameter. Patients with multiple scans were recorded. Probe type, operator, IQR/median stiffness ratio and number of valid measurements were also recorded. Laboratory scoring systems commonly used in FALD, the Fibrosis-4 (FIB-4) score and aspartate aminotransferase to platelet ratio index (APRI) were noted from either the TE report or calculated from valid laboratory tests. Tests were deemed valid if they were reported within three months of the TE scan.

Biochemistry results were collated if conducted within 12 months from a TE scan, including albumin, alkaline phosphatase, alanine aminotransferase, aspartate aminotransferase (AST), gamma-glutamyl transferase (GGT), bilirubin, platelet count, international normalised ratio and alpha fetoprotein, These results were collated from stable, outpatient settings if available, otherwise were sourced from periods of stable disease. Results of other testing such as ultrasound imaging, or liver biopsy were noted if conducted within 12 months of the TE scan.

Haemodynamic and functional performance of the Fontan circulation was evaluated from echocardiography, cardiac magnetic resonance imaging and cardiac catheterisation reports and deemed valid if performed within a year of TE reports. Echocardiogram information collected included inferior vena cava (IVC) diameter, ejection fraction, IVC to Fontan flow and pulmonary artery flow. The ventricular dysfunction and degree of atrioventricular regurgitation in the dominant systemic ventricle were graded as preserved, mild, moderate or severely reduced. Cardiac magnetic resonance imaging parameters include Fontan conduit size divided by body surface area, dominant ventricle end diastolic volume divided by body surface area, dominant ventricle end systolic volume divided by body surface area, dominant ventricle ejection fraction and IVC arterial flow divided by body surface area. Cardiac catheterisation data collected include mean Fontan pressure, dominant ventricle end diastolic pressure and pulmonary artery pressure.

### 2.3 Endpoints and derived secondary outcomes

The primary endpoints assessed were (a) features of decompensated cirrhosis defined by the presence of ascites, variceal haemorrhage or overt hepatic encephalopathy [8] and (b) abnormal imaging deemed significant enough to warrant hepatocellular carcinoma multi-disciplinary team meeting.

Clinically significant portal hypertension (CSPH) was assessed using the Baveno VII criteria. Patients with an elevated risk of CSPH were defined as having LSM values of 15-20 kPa and platelet count <110 × 10^9^/L, 20-25 kPa and platelet count <150 × 10^9^/L or LSM values ≥ 25 kPa as per the Baveno VII guidelines [8]. However, as LSM forms part of the Baveno VII definition, CSPH was analysed as a derived surrogate outcome rather than an independent endpoint.

### 2.4 Data analysis

Data is represented as count and percentage for categorical data and as median and interquartile range (IQR) for continuous data. A linear mixed effects model was used to compare serum biomarkers and TE measurements between patients with right and left dominant ventricles, to account for repeated scans across some patients. Generalised estimating equation regression analyses were also employed for selected analyses to provide population-averaged estimates whilst accounting for the multiple TE scans some patients received. This was used to analyse the trend in median LSM with each increasing decade post-Fontan surgery, to assess whether patients with multiple TE scans recorded any significant trend and to assess the statistical correlation between median LSM and laboratory markers. Correlation between cardiac parameters and LSM values were analysed for variables with more than ten reports. A linear mixed effects model was utilised to adjust for multiple echocardiogram reports for a single patient, otherwise the Kruskal Wallis test was utilised. The proportion of patients as stratified by decade year that developed CSPH was analysed using the Pearson's Chi-squared test. Furthermore, correlation between laboratory parameters and CSPH were analysed using a logistic regression model. For all statistical analyses, a significant result was defined to be p < 0.05. All analyses were performed on R studio Version 2024.04.2 + 764, and all data was stored on Excel Version 16.94.

## 3 Results

### 3.1 Study population

There were a total of 65 adult post-Fontan patients with baseline characteristics presented in Table 1. All Fontan patients under specialist cardiac care were referred for hepatology follow up. The decision to proceed with transient elastography testing was driven by clinical suspicion rather than a set protocol. A total of 111 Fibroscans™ were performed over the study period. The median IQR/median stiffness ratio was 0.11 (IQR: 0.08-0.145). The majority of Fibroscans™ were performed using the medium sized probe (n = 87, 78%) with the remaining scans obtained using the extra-large probe (n = 24, 21%). Scan quality was standardised, with all examinations deemed successful or valid by the operator and reporting physician. Each Fibroscan™ report included at least ten valid measurements, and all scans were performed by trained operators at the same tertiary centre. No invalid scans were included. There were more males (52%) and the median age at presentation was 28 (IQR: 26-33) years. The median age at Fontan completion was 4 years (IQR: 3-6). 39% had a morphologic left ventricle, 39% had a morphologic right ventricle and 22% were indeterminate. After adjusting for age, patients with right-dominant ventricles had slightly lower albumin (−2.85 g/L, 95% CI: −5.90 – 0.20, p = 0.07) and slightly higher ALP (17.0 U/L, 95% CI: −1.98 – 36.1, p = 0.08) compared to those with left-dominant ventricles, although this was not significant. No differences were observed for other serum biomarkers including ALT, AST, GGT, bilirubin, platelet count, INR or median LSM between left and right morphologic ventricles. Fifty-one patients (78%) received an extracardiac conduit as their Fontan technique. Twelve patients received a lateral tunnel technique and two patients had a atriopulmonary Fontan procedure. Notably the two atriopulmonary Fontan patients had their Fontan operation in the early 1990s.

**Table 1:** Demographic and baseline characteristics of adult post-Fontan surgery patients.

Cohort size	65
Age (years)	28 (26-33)
Sex	
Males	34 (52%)
Females	31 (47%)
Age at Fontan (years)	4 (3-6)
Primary morphological diagnosis	
Tricuspid atresia	9 (14%)
Hypoplastic left heart syndrome	9 (14%)
Double outlet right ventricle	10 (15%)
Double inlet left ventricle	11 (17%)
Transposition of the great arteries	8 (12%)
Atrioventricular septal defect	3 (5%)
Pulmonary atresia with intact ventricular septum	5 (8%)
Other	7 (11%)
Unknown	3 (5%)
Fontan Type	
Extracardiac conduit	51 (78%)
Lateral tunnel	12 (18%)
Atriopulmonary	2 (3%)
Medication	
Warfarin	11 (17%)
Aspirin	40 (62%)
Angiotensin-converting enzyme inhibitor	15 (23%)
Aldosterone antagonists	4 (6%)
Beta-blockers	11 (17%)
Contraceptive agents	3 (5%)
BMI	
<18.5	1 (2%)
18.5-24.9	35 (54%)
25.0-29.9	14 (22%)
30.0-34.9	7 (11%)
35.0-39.9	2 (3%)
>40.0	0 (0%)
NA	6 (9%)
Alcohol consumption (mild)	2 (3%)

Data is represented as median (IQR) or n (%).

### 3.2 Fibroscan™ data

The median time from Fontan surgery to the first Fibroscan™ was 17 years (IQR: 15-23) and the median LSM for the first Fibroscan™ was 15.6 kPa (IQR: 12.6-19.5). The median controlled attenuation parameter was 226 (IQR: 194-274).

**Fig. 1:**
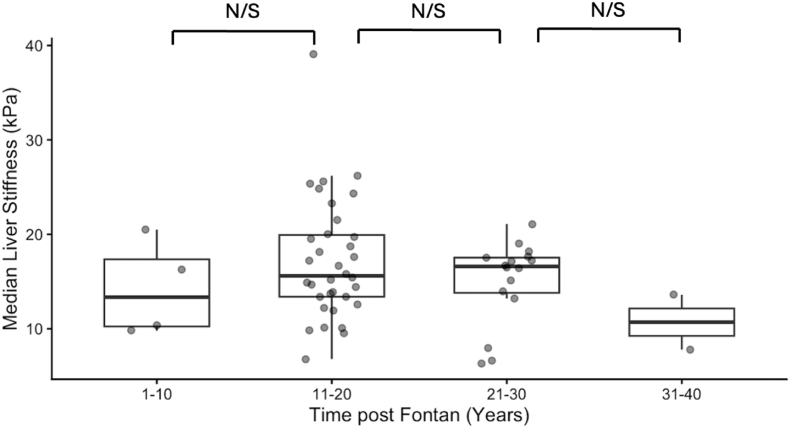
Box and whisker plots identifying median and interquartile ranges for median liver stiffness measurement as measured by transient elastography for each decade-year post-surgery. Significance was analysed using a general estimating equation to assess median liver stiffness measurements (kPA) between two consecutive decade-years post-Fontan surgery. Significance was set at p < 0.05. Grey dots represent each individual Fibroscan™ measurement datapoint. Abbreviations: N/S = not significant.

For each patient's first Fibroscan™, there was no significant increase in median LSM between the first (1-10 years) and the second decade (11-20 years) post-Fontan surgery (p = 0.40, Fig. 1). There was also no significant change between the second to third decade (21-30 years) or the third to fourth decade (31-40 years) post-Fontan (p = 0.50, 0.20 respectively, Fig. 1). Notably, the patient with the highest median LSM (39.1 kPa) was 17 years post-Fontan surgery and the patient with the longest post-Fontan timeframe (33 years) recorded a median LSM of 7.8 kPa.

**Fig. 2:**
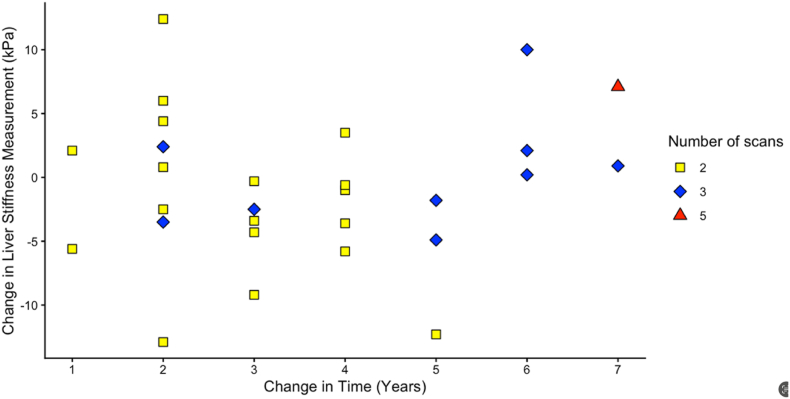
Scatter plot depicting the change in median liver stiffness measurements (kPA) with respect to the change in time (years) between the first and last Fibroscan™ for patients that had multiple scans. For example, the red triangle in the graph represents the patient who had five Fibroscans™. The difference between their first and last median LSM value is 7.1 kPA and the time elapsed from their first Fibroscan™ to their last Fibroscan™ was seven years. Scans with incomplete data (n = 10) on Fontan surgery date were excluded.

There was no significant trend for patients that had multiple Fibroscans™. Fig. 2 describes the change in median LSM values when comparing a patient's last measurement to their first measurement. Fourteen patients reported an increase in LSM, and 19 patients reported a decrease in LSM. Patients with two Fibroscans™ reported a median change of −1.6 kPa (IQR: −4.175-1.825, n = 22). Patients with three scans reported a median change in LSM between their first and last scan of −0.8 kPa (IQR: −3.25-1.8, n = 10). Finally, one patient had five scans and their difference in LSM was 7.1 kPa. There was no relationship between time elapsed from the first scan to the last scan and LSM changes (p = 0.50, Fig. 2).

Eighteen patients had a significant change in stiffness, defined as a change of 20% or more when comparing final to initial LSM values. Of these patients, eight reported an increase in LSM with a median increase of 19% per annum, whereas ten patients reported a median decrease in LSM of 10% per annum.

### 3.3 Laboratory markers

Table 2 describes the relationship between blood parameters and median LSM for all 111 scans. A generalised estimating equation analysis was used to account for the multiple Fibroscans™ some patients received. Increasing ALT, international normalised ratio, AST and GGT all correlated with increasing median LSM (p = 0.036, <0.001, <0.001, <0.001 respectively). An increasing median LSM also correlated with decreasing platelet count (p = 0.027). The APRI and FIB-4 both correlated with median LSM values (p < 0.001, p < 0.001).

**Table 2:** Generalised estimating equation analysis analysing the relationship between serology markers and laboratory scoring systems to median liver stiffness measurements.

Serology	Median (IQR)	p-value	Serology	Median (IQR)	p-value
Platelets (x10^9^)	196 (154-241)	**0.027**	INR	1.2 (1.1-1.37)	**<0.001**
Albumin (g/L)	45 (42-46)	0.09	AST (U/L)	27 (22-33)	**<0.001**
ALP (U/L)	75 (66-94)	0.32	GGT (U/L)	64 (40-84)	**<0.001**
Bilirubin (μmol/L)	17.5 (11.2-24.8)	0.13	ALT (U/L)	31.5 (24-41)	**0.036**
FIB-4	0.62 (0.443-0.85)	**<0.001**	APRI	0.4 (0.3-0.5)	**<0.001**

Statistical significance was set at p < 0.05. Abbreviations: IQR = interquartile range, ALP = alkaline phosphatase, ALT = alanine aminotransferase, AST = aspartate aminotransferase, GGT = gamma-glutamyl transferase, INR = international normalised ratio, FIB-4 = fibrosis-4, APRI = Aspartate aminotransferase to platelet index.

### 3.4 Cardiac parameters

A total of 85 echocardiograms were performed within one year of the corresponding Fibroscan™ for all TE reports; however three echocardiograms did not have any required data points for analysis, resulting in 82 echocardiograms. A linear mixed effects model was employed for statistical analysis if multiple echocardiogram parameters were available for a given patient, otherwise the Kruskal Wallis test was used. Among these, eleven reports included the IVC diameter [median (IQR): 19 (16.35-20.27) mm]. A larger IVC diameter was significantly associated with higher median LSM values (p = 0.032, Table 3). Only two echocardiograms included ejection fraction. Ventricular function was preserved in most echocardiograms (n = 70, 85.37%) with mild, moderate and severe dysfunction observed in 10 (12.20%), 1 (1.22%) and 1 (1.22%) scan respectively. The degree of ventricular function correlated significantly with median LSM values (p < 0.001). Most scans had no or mild atrioventricular valve regurgitation in the dominant ventricle [n = 23 (28.05%) and n = 51 (62.20%), respectively, Table 3]. Normal IVC to Fontan flow and normal pulmonary artery flow [n = 79 (96.34%), n = 80 (97.56%) respectively] was seen in most patients. Degree of atrioventricular regurgitation, IVC to Fontan flow and pulmonary artery flow were not associated with median LSM (p = 0.42, p = 0.12, 0.73 respectively, Table 3).

**Table 3:** Haemodynamic and functional assessment of the Fontan circulation.

	Median (IQR) or n (%)	n	p-value
Echocardiogram			
IVC diameter (mm)	19 (16.35-20.27)	11	**0.032**
EF (%)	56 (55-57)	2	NA
Ventricular function		82	**<0.001**
Preserved	70 (85.37%)		
Mildly reduced	10 (12.20%)		
Moderately reduced	1 (1.22%)		
Severely reduced	1 (1.22%)		
AV valve regurgitation		82	0.42
None	23 (28.05%)		
Mild	51 (62.20%)		
Moderate	8 (9.76%)		
Severe	0 (0%)		
IVC flow to Fontan		82	0.12
Normal	79 (96.34%)		
Obstructed	3 (3.66%)		
Pulmonary artery flow		82	0.73
Normal	80 (97.56%)		
Obstructed	2 (2.44%)		
**Cardiac MRI**			
Dominant ventricle EDV/BSA (mL/m^2^)	85.57 (67.91-95.79)	9	NA
Dominant ventricle ESV/BSA (mL/m^2^)	33.83 (30.86-52.63)	9	NA
Dominant ventricle EF (%)	52.5 (50.25-60.25)	12	0.81
IVC arterial flow/BSA (L/min/m^2^)	1.88 (1.59-2.12)	5	NA
**Cardiac Catheterisation**			
Mean Fontan pressure (mmHg)	15 (9.5-17.5)	7	NA
Dominant ventricle end diastolic pressure (mmHg)	14.5 (12.5-18)	8	NA
Pulmonary artery pressure (mmHg)	15 (11-17)	9	NA

Significance testing was conducted to analyse the relationship between variables with more than ten results and median liver stiffness measurement as measured through transient elastography. A linear mixed effects model was utilised when there were multiple echocardiogram reports for the same patient for a given variable, otherwise the Kruskal Wallis test was applied.

Abbreviations: IQR = interquartile range, n = number, IVC = inferior vena cava, EF = ejection fraction, AV = atrio-ventricular, MRI = magnetic resonance imaging, EDV = end diastolic volume, ESV = end systolic volume, BSA = body surface area.

Twelve cardiac magnetic resonance imaging reports were conducted within a year of all Fibroscan™ analysed. No reports included the Fontan conduit/body surface area value. Median end diastolic volume/body surface area and end systolic volume/body surface area for the dominant ventricle was 85.57 mL/m^2^ (IQR: 67.91-95.79, n = 9) and 33.83 mL/m^2^ (IQR: 30.86-52.63, n = 9) respectively. Median ejection fraction was 52.5% (IQR: 50.25-60.25, n = 12, Table 3) and was not associated with median LSM (p = 0.81).

Twelve cardiac catheterisations were conducted within a year of all Fibroscans™, however significance testing was not performed due to missing data. Median Fontan pressure was 15 mmHg (IQR: 9.5-17.5, n = 7, Table 3), median dominant ventricle end-diastolic pressure was 14.5 mmHg (IQR: 12.5-18, n = 8, Table 3) and median pulmonary artery pressure was 15 mmHg (IQR: 11-17, n = 9, Table 3).

### 3.5 Endpoints

#### 3.5.1 Decompensated cirrhosis

Four patients exhibited features of decompensated cirrhosis (n = 4, 6%), with three having significant ascites and one with Grade 2 varices on endoscopy. These patients did not have other causes of acute hepatic decompensation, including viral hepatitis, hemochromatosis or alcohol related liver disease. Of these four patients, no patients were obese or were recently started on hepatotoxic medication that could explain the acute decompensation. Three patients received an extracardiac Fontan operation whereas one received a lateral tunnel variant. Patients with decompensated cirrhosis had a median post Fontan surgery time of 16.5 years (IQR: 15-18.25) and had a median LSM value of 16.8 kPa (IQR: 15.13-18.9) on their first elastography scan at time of decompensation. Patients without features of decompensated cirrhosis were summarised using their first scan and had a median LSM value of 15.4 kPa (IQR: 12.6-19) and were a median 17 years (IQR:15-23) post-Fontan surgery. Of the patients with decompensated cirrhosis, one died from end stage cardiac disease, and another was diagnosed with hepatocellular carcinoma. There was no significant difference in first TE LSM values between those that did or did not develop decompensated cirrhosis (p = 0.30). Furthermore, each unit of kPa increase in LSM was associated with 3.8% increased odds of decompensated cirrhosis (OR: 1.04, 95% CI: 0.97-1.11), with the non-significance likely reflecting the small sample size rather than a true absence of association. Steatosis measurements were assessed using median controlled attenuation parameters (CAP) on TE for each scan. Patients that did eventually decompensate had a median CAP of 218 dB/m (IQR: 217.5-240) whereas patients that did not decompensate had a median CAP of 229 dB/m (IQR: 193-278). However, this was not significant [OR: 1.00 (1.00-1.01), p = 0.20].

Early prediction of decompensation (EPOD) scores were calculated for the first scan of each patient to assess the difference in scores between patients that did not and did ultimately decompensate. The median EPOD score for the first scan of each patient that did compared to patients that did not decompensate was 11.31 (IQR:8.75-13.71) and 9.31 (IQR: 8.63-10.43) respectively. This was not statistically significant (p = 0.40).

#### 3.5.2 Hepatocellular carcinoma multi-disciplinary team meetings

Eight patients warranted a hepatocellular carcinoma multi-disciplinary team discussion as they had suspicious lesions on imaging (12%). One patient had hepatocellular carcinoma. This patient also had clinical features of decompensated cirrhosis and was 15 years post Fontan with a LSM value of 10.1 kPa and CAP of 218 dB/m. From the seven remaining patients, four patients had focal nodular hyperplasia, and three had lesions deemed non-specific and non-concerning, with stability over time.

### 3.6 Derived surrogate outcomes

#### 3.6.1 CSPH

**Fig. 3:**
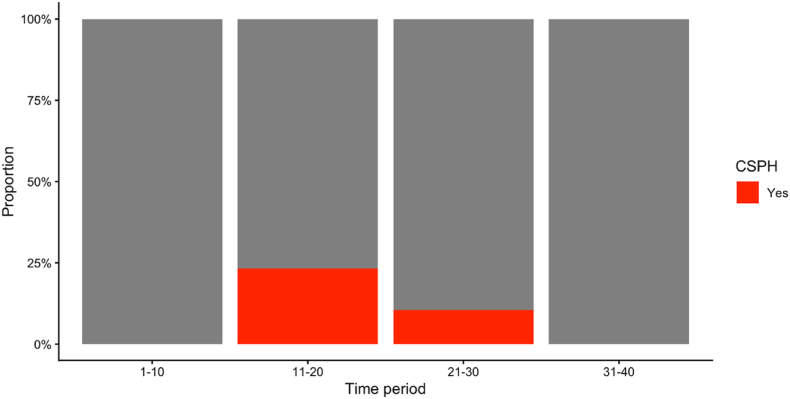
Proportion of patients with clinically significant portal hypertension as stratified by time post Fontan surgery. Clinically significant portal hypertension is defined as per the Baveno VII guidelines. A significant difference in the proportion ratios between time groups was observed (p < 0.001), as determined by Pearson's Chi-squared test. Abbreviations: CSPH = clinically significant portal hypertension.

Ten scans reported a LSM ≥25, five scans reported a combination of LSM value of 20-25 kPa and platelet count <150 × 10^9^/L, and no scans reported a LSM value of 15-20 kPa and platelet count <110 × 10^9^/L. This corresponded to 15 scans across 12 unique patients (18%) who fulfilled the Baveno VII diagnosis of CSPH. There was a significant association between Fontan type (extracardiac, lateral tunnel and atriopulmonary) and developing CSPH (p < 0.001) with the atriopulmonary group having the highest proportion of patients with CSPH, although this is likely confounded by small sample size. Fig. 3 illustrates the proportion of patients with CSPH, categorised by their respective time groups post-Fontan surgery. No patients 1-10 years post-Fontan or 31-40 years post-Fontan had CSPH. Patients with complete biochemical markers were analysed to assess which serology markers were associated with the development of CSPH. Higher AST [OR (95% CI): 1.06 (1.01-1.14), p = 0.049], GGT [OR (95% CI): 1.01 (1.00-1.03), p = 0.047], bilirubin [OR (95% CI): 1.06 (1.01-1.12), p = 0.034] and INR values [OR (95% CI): 10.04 (2.05-81.73), p = 0.012], were significantly associated with increased likelihood of developing CSPH.

## 4 Discussion

This study demonstrates a high prevalence of both clinical and surrogate indices of liver damage associated with the Fontan circulation. After a median of 19 years post-Fontan, almost 20% of patients were diagnosed with CSPH as assessed by TE and platelet count. There was no trend between a patient's first recorded LSM value and time post-Fontan procedure or episodes of decompensated cirrhosis. However, this should be interpretated with caution as only four patients exhibited features of decompensated cirrhosis, and one had hepatocellular carcinoma. This small sample size likely precludes clinically meaningful LSM interpretation in the decompensated cohort and non-significant results cannot definitively be interpretated as having no association. However, increasing LSM scores did correlate significantly with larger IVC diameters and ventricular dysfunction on echocardiogram. Eight patients had lesions suspicious enough to warrant a multi-disciplinary team discussion, with one having confirmed hepatocellular carcinoma.

FALD is infrequently seen by hepatologists, however, given the often prolonged survival of patients, liver manifestations are being increasingly recognised [2,11]. Like this study, the literature reports that close to two-thirds of patients have sonographic evidence of structural hepatic damage [12]. Whilst not commonly performed, bridging fibrosis has been reported in over one-third of patients within the first decade post-Fontan through liver biopsy [13]. Furthermore, the annual incidence of hepatocellular carcinoma in FALD patients is reported as 1.3% although significant heterogeneity has been reported [5,11]. In a 2025 study conducted by Janzen et al., almost 40% of patients experienced at least one of congestive heart failure, cardiac hospitalisation, thrombosis, protein losing enteropathy or death in a six year follow-up time period [9]. In the new clinical guidelines released by the American College of Cardiology in late 2025, adults with Fontan circulation are recommended to undergo hepatic screening by experienced hepatologists with imaging at least annually [14]. Taken together, this data makes the case for standardised long-term surveillance protocols with management algorithms formed with serial good quality data from experienced centres [5].

There is currently no standard-of-care model for FALD. Liver biopsy as traditionally undertaken in cases of clinical uncertainty, is risky due in part to the long-term anticoagulation some of these patients are on [7,13]. While non-invasive techniques, such as used in this study, form an alternative, the literature remains divided on their utility. Some studies report a poor correlation between LSM and hepatic decompensation, whereas others suggest that they are valuable in evaluating FALD [12,[15], [16], [17], [18]]. In this study, TE did not correlate with decompensating events or hepatocellular carcinoma development. Median LSM did not significantly change with time post Fontan surgery, suggesting that there is significant heterogeneity in clinical presentation and progression regardless of time post-surgery. Furthermore, only one patient developed hepatocellular carcinoma in this study, and their LSM was 10.1 kPa, lower than the median LSM of all patients (15.8 kPa), and lower than the threshold that would suggest cirrhosis and mandate routine surveillance for hepatocellular carcinoma in other contexts [19,20].

The inability of TE to accurately predict adverse liver outcomes may be related to variable contributions from hepatic venous congestion from reduced venous return due to altered cardiac physiology [17,21]. This may reflect the disordered blood flow during the disease course, which ultimately increases the risk of parenchymal ischaemia and extinction, leading to adverse clinical outcomes [22]. Minimal correlation was also seen between echocardiogram parameters and TE in this study, suggesting that LSM and echocardiogram data cannot be solely relied upon when evaluating FALD. Rather, risk stratification algorithms need to be established in multi-centre studies to better capture high-risk individuals before the onset of irreparable hepatic damage.

In our study, laboratory markers significantly associated with both CSPH and median LSM were GGT, INR and AST. This aligns with the literature which reports elevated GGT levels as the most common finding in FALD [5,23]. The GGT elevation is particularly noteworthy as it is more likely to be related to systemic oxidative stress in the context of a Fontan circulation and is known to be associated with long-term adverse liver outcomes in large scale population studies [5,24,25]. Indeed, GGT elevations are one of the best predictors of liver mortality [26,27] and should be routinely tested for early diagnosis of liver disease [28]. These markers can be combined in scoring systems; however, none have been validated specifically for FALD [29]. Studies attempting to develop scoring systems were either limited by small cohort size (n = 79) [30], or were conducted on a skewed population distribution [1,29].

Laboratory scoring systems as surrogates for liver fibrosis are not validated in FALD but often used include APRI and FIB-4 [5]. In this study, APRI and FIB-4 was significantly associated with TE (p < 0.001, p < 0.001) [6]. The utility of APRI and FIB-4 in FALD is supported by other studies however they only have modest discriminatory powers in identifying adults with advanced liver disease [13,31]. Again, this highlights the variable contributions to non-invasive assessments by congestion, architectural distortion and fibrosis [17,21]. We suggest that larger multi-institutional collaborative studies with larger sets of baseline variables without *a priori* inclusion of variables based on other liver diseases is required, including novel serum based markers such as Pro-C3, ADAPT and ELF, and exploitation of artificial intelligence [32]. This will help define novel scores that predict disease stage and clinical outcomes in FALD.

A more nuanced scoring system would also incorporate cardiovascular risk factor including Fontan technique, echocardiogram and cardiac MRI results. In this study, the limited data for echocardiogram and cardiac MRI results restrict the statistical power of cardiac-hepatic analyses and should only be interpreted as exploratory. Indeed, Fontan type may influence the development of FALD due to atrial and venous haemodynamic differences [33]. Atriopulmonary Fontan is associated with greater atrial dilation, energy losses and venous congestion whereas the more recent extracardiac conduit has improved haemodynamic performance [33]. However, FALD has been reported across all Fontan types, suggesting that other risk factors also warrant consideration [34]. These include, but are not limited to, degree of ventricular dysfunction, atrio-ventricular valve dysfunction, fenestration presence and pulmonary capillary wedge pressure [11,35].

There were limitations to this study. First, liver biopsy was not available for most patients, so TE could not be compared against the gold standard. Echocardiograms were also not systematically collected at the time of TE due to the retrospective nature of the study which would have enabled a more nuanced interpretation of elastography values. Furthermore, CSPH in this study was defined using the Baveno VII criteria which incorporates LSM within the definition; therefore, CSPH represents a derived surrogate outcome rather than an independent clinical endpoint. These thresholds have also not been validated in the Fontan population, where hepatic congestion may elevate liver stiffness independent of fibrosis, potentially limiting the interpretation of the CSPH classification. This combined with the single-centre nature of the study limits the statistical power and generalisability of the results to the broader Fontan population.

In conclusion, our study highlights the need for long term liver surveillance post-Fontan surgery. While non-invasive technologies such as transient elastography and laboratory markers demonstrate some association with surrogate markers of liver disease, their predictive value for clinical outcomes remains uncertain. In this study, the limited sample size likely contributed to the absence of significant outcomes, however, the findings add to the growing body of research in this rare group of individuals. Further research is needed, ideally without preconceived notions about variable selection along with the inclusion of cardiac parameters to identify specific predictive factors and their thresholds for effective risk stratification prior to the onset of irreversible hepatic disease. Good quality serial measurements and regular testing may improve the ability to detect early changes in hepatic function and fibrosis progression, allowing for timely intervention before the onset of irreversible liver disease.

## Funding

JG is supported by the following grants: the Robert W. Storr Bequest to the Sydney 10.13039/100001236Medical Foundation, University of Sydney; a 10.13039/501100000925National Health and Medical Research Council of Australia Program Grant (APP1053206), Investigator and MRFF grants (APP2032407; NCRI000183; APP2016215; APP 2010795; APP1196492) and a 10.13039/501100001171Cancer Institute NSW grant (2021/ATRG2028). No grants had any involvement in the study design, collection, analysis and interpretation of data, writing of the report or decision to submit the article for publication.

## CRediT authorship contribution statement

**Louise Cai:** Data curation, Formal analysis, Investigation, Methodology, Writing – original draft, Writing – review & editing. **Preeti Choudhary:** Writing – review & editing. **David Tanous:** Writing – review & editing. **Jacob George:** Conceptualization, Funding acquisition, Project administration, Supervision, Visualization, Writing – original draft, Writing – review & editing.

## Declaration of competing interest

The authors declare the following financial interests/personal relationships which may be considered as potential competing interests: Jacob George reports financial support was provided by Sydney Medical School Foundation. Jacob George reports financial support was provided by National Health and Medical Research Council. Jacob George reports financial support was provided by Investigator and MRFF. Jacob George reports financial support was provided by Cancer Institute NSW. If there are other authors, they declare that they have no known competing financial interests or personal relationships that could have appeared to influence the work reported in this paper.

## Data Availability

The data that support the findings of this study are available upon reasonable request from the corresponding author. The data are not publicly available due to privacy or ethical restrictions.
